# Enhanced airway hyperresponsiveness in asthmatic children and mice with A(H1N1)pdm09 infection

**DOI:** 10.1002/iid3.406

**Published:** 2021-01-20

**Authors:** Taira Ariyoshi, Junichiro Tezuka, Hiroki Yasudo, Yasufumi Sakata, Tamaki Nakamura, Takeshi Matsushige, Hideki Hasegawa, Noriko Nakajima, Akira Ainai, Atsunori Oga, Hiroshi Itoh, Komei Shirabe, Shoichi Toda, Ryo Atsuta, Shouichi Ohga, Shunji Hasegawa

**Affiliations:** ^1^ Department of Pediatrics Yamaguchi University Graduate School of Medicine Yamaguchi Japan; ^2^ Department of Allergy and Pulmonology Fukuoka Children's Hospital Fukuoka Japan; ^3^ Department of Pediatrics National Hospital Organization Fukuokahigashi Medical Center Fukuoka Japan; ^4^ Department of Pathology National Institute of Infectious Diseases Tokyo Japan; ^5^ Department of Molecular Pathology Yamaguchi University Graduate School of Medicine Yamaguchi Japan; ^6^ Yamaguchi Prefectural Institute of Public Health and Environment Yamaguchi Japan; ^7^ Akihabara Atsuta Clinic Tokyo Japan; ^8^ Department of Pediatrics, Graduate School of Medical Sciences Kyushu University Fukuoka Japan

**Keywords:** airway resistance, influenza, pandemic, respiratory function test

## Abstract

**Background:**

Severe asthma exacerbation is an important comorbidity of the 2009 HIN1 pandemic (A(H1N1)pdm09) in asthmatic patients. However, the mechanisms underlying severe asthma exacerbation remain unknown. In this study, airway hyperresponsiveness (AHR) was measured in pediatric asthma patients infected with A(H1N1)pdm09. We also evaluated AHR in asthmatic mice with A(H1N1)pdm09 infection and those with seasonal influenza for comparison.

**Methods:**

AHRs in asthmatic children were defined as the provocative acetylcholine concentration causing a 20% reduction in forced expiratory volume in 1 s (PC_20_). To investigate the pathophysiology using animal models, BALB/c mice aged 6‐8 weeks were sensitized and challenged with ovalbumin. Either mouse‐adapted A(H1N1)pdm09, seasonal H1N1 virus (1 × 10^5^ pfu/20 μl), or mock treatment as a control was administered intranasally. At 3, 7, and 10 days after infection, each group of mice was evaluated for AHR by methacholine challenge using an animal ventilator, flexiVent. Lung samples were resected and observed using light microscopy to assess the degree of airway inflammation.

**Results:**

AHRs in the children with bronchial asthma were temporarily increased, and alleviated by 3 months after discharge. AHR was significantly enhanced in A(H1N1)pdm09‐infected asthmatic mice compared to that in seasonal H1N1‐infected mice (*p* < .001), peaking at 7 days postinfection and then becoming similar to control levels by 10 days postinfection. Histopathological examination of lung tissues showed more intense infiltration of inflammatory cells and severe tissue destruction in A(H1N1)pdm09‐infected mice at 7 days postinfection than at 10 days postinfection.

**Conclusion:**

Our results suggest that enhanced AHR could contribute to severe exacerbation in human asthmatic patients with A(H1N1)pdm09 infection.

## Graphical abstract

AHR was significantly enhanced in asthmatic mice with A(H1N1)pdm09 infection compared with seasonal influenza infection. AHR and pulmonary inflammation of A(H1N1)pdm09‐infected mice showed peak at 7 days post infection and they were improved by 10 days postinfection. Also, AHR in asthmatic children after A(H1N1)pdm09 infection were temporarily increased, and alleviated by 3 months after discharge.

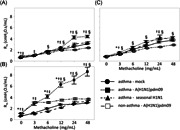

## 1 INTRODUCTION

Asthma exacerbation is a major cause of disease morbidity that increases health care costs, and in some patients, progressive loss of lung function.^1^ Exposure to aeroallergens and environmental factors, such as smoking, PM2.5, or diesel exhaust particles, triggers asthma exacerbation.^2^, ^3^ Respiratory viral infection is also associated with the pathophysiology of asthma exacerbation, particularly in childhood.^4^ The most prominent pathogens involved in asthma exacerbation include human rhinovirus, respiratory syncytial virus, enterovirus, influenza virus, and human metapneumovirus.^5^, ^6^, ^7^

Many severe and fatal cases of the 2009 HIN1 pandemic (A(H1N1)pdm09) infection have been reported, both in patients with underlying diseases as well as in healthy children and young adults.^8^, ^9^, ^10^ Asthma is among the most common underlying conditions in patients hospitalized with A(H1N1)pdm09 infection and asthmatic children show greater susceptibility to A(H1N1)pdm09 viral infection,^11^ suggesting that severe asthma exacerbation is an important comorbidity of influenza infection in patients with asthma.^8^, ^9^, ^10^

We previously reported that A(H1N1)pdm09 infection, but not seasonal H1N1 infection, induces severe pulmonary inflammation with elevated cytokine levels in a mouse model of asthma.^12^, ^13^ Moreover, asthmatic model mice with A(H1N1)pdm09 infection are prone to an earlier onset of severe pulmonary inflammation compared to those with seasonal H1N1 infection,^14^ suggesting that a hyper‐cytokine condition is involved in severe pneumonia and atelectasis. Another report showed that A(H1N1)pdm09 induces AHR in a non‐asthmatic mouse model.^15^ However, no reports have evaluated the effect of A(H1N1)pdm09 on airway constriction in patients with asthma and, to date, the mechanisms of severe asthma exacerbation due to A(H1N1)pdm09 infection remain unclear.

In this study, we showed that pediatric patients with A(H1N1)pdm09 infection temporarily increased airway hyperresponsiveness (AHR) as well as asthmatic mice. Furthermore, we showed that the changes in AHR are greater in asthmatic mice with A(H1N1)pdm09 infection than those with seasonal H1N1 influenza. These findings indicate that enhanced AHR contributes to the severe asthma exacerbation phenotype triggered by A(H1N1)pdm09 infection.

## 2 METHODS

### 2.1 Measurement of AHR in children with A(H1N1)pdm09 infection

Studies on pediatric patients were carried out in accord with the Declaration of Helsinki. This study enrolled pediatric asthmatic patients with A(H1N1)pdm09 infection and hypoxia (SpO_2_ ≤ 90%) within the first day of fever admitted to Fukuokahigashi Medical Center between September 2009 and December 2010. Bronchial asthma was diagnosed and the severity was classified according to the Japanese Pediatric Guidelines for the Treatment and Management of Bronchial Asthma 2008 (JPGL 2008).^16^ The participants were diagnosed with A(H1N1)pdm09 infection by polymerase chain reaction at Fukuoka Institute of Health and Environmental Sciences. AHRs were measured at 1 and 3 months after discharge. Anti‐inflammatory drugs and bronchodilators were prohibited 1 month after discharge. Increasing concentrations of acetylcholine (39, 78, 156, 312, 625, 1,250, 2,500, 5,000, 10,000, and 20,000 μg/ml) were inhaled by a nebulizer until the forced expiratory volume in 1 s (FEV_1.0_) was reduced by 20% from a post‐nebulized saline value. FEV_1.0_ was measured using a spirometer (HI‐801, CHEST M.I., Inc., Tokyo, Japan). AHR was defined as the provocative concentration causing a 20% fall in FEV_1.0_ (PC_20_). This study was approved by the institutional review board of Fukuokahigashi Medical Center (2020‐rin‐8). Parents were explained this study and signed informed consent.

### 2.2 Sensitization and allergen challenge in a mouse model of bronchial asthma

BALB/c mice aged 6–8 weeks were obtained from Chiyoda Kaihatsu Co. (Tokyo, Japan) and sensitized and challenged with grade II ovalbumin (Sigma‐Aldrich., St. Louis, MO) as previously described.^12^, ^13^, ^14^ All animal experimentation procedures were approved by the Institutional Animal Care and Use Committee of Yamaguchi University (No. 29‐S01), and all methods were conducted in accordance with approved guidelines. Additionally, animal experiments conformed to the revised Institute of Laboratory Animal Resources, Commission on Life Sciences, National Research Council “Guide for the Care and Use of Laboratory Animals” published by the National Academy Press, Washington, D.C. 1996.

### 2.3 Virus infection

Mouse‐adapted A(H1N1)pdm09 (strain: A/Narita/1/09) or seasonal H1N1 (strain: A/Puerto Rico) viruses were provided by the National Institute of Infectious Diseases (Tokyo, Japan). On Day 31, influenza virus (concentration: 1 × 10^5^ pfu/20 μl) or vehicle (mock‐infection) was administered intranasally to the mice. The number of mice in each group ranged from 4 to 7.

### 2.4 Measurement of AHR

Mice were anesthetized by intraperitoneal injection of xylazine (12 mg/kg) and pentobarbital (70 mg/kg) at 3, 7, and 10 days postinfection. After anesthetization, mice were cannulated and connected to an animal ventilator (flexiVent, SCIREQ, Montreal, QC, Canada). The mice inhaled aerosolized phosphate‐buffered saline or 3, 6, 12, 24, or 48 mg/ml methacholine in phosphate‐buffered saline. We measured the following parameters: resistance of the respiratory system (R_rs_) and tissue damping (G); R_rs_ and G reflect the resistance of the total and peripheral airways, respectively.^17^, ^18^ These parameters were measured at frequent intervals of 10–15 s 12 times following activation of the nebulizer.

### 2.5 Histological examination of the lungs

After the methacholine challenge, the lung was resected from mice that were euthanized with high dose pentobarbital (200 mg/kg). Lung tissues were fixed in 10% buffered formalin for 24 h at room temperature and then embedded in paraffin. Serial sections (3 μm‐thick) were cut and stained with hematoxylin and eosin (Muto Pure Chemicals Co., Tokyo, Japan).

### 2.6 Statistical analysis

For human data, differences between groups were analyzed using the Wilcoxon signed rank test. For mouse data, differences between groups were analyzed using the Steel–Dwass test. When *p* values less than .05, differences between means were considered to be statistically significant. All analyses and calculations were performed using JMP® Pro version 13.0.0 software (SAS Institute, Inc., Cary, NC).

## 3 RESULTS

### 3.1 Measurement of AHR in children with A(H1N1)pdm09 infection

**Figure 1:**
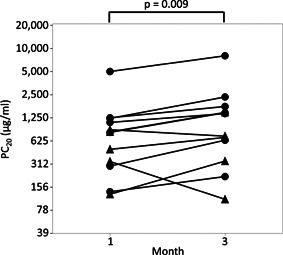
The provocative concentration causing a 20% reduction in the forced expiratory volume in 1 s (PC_20_) at 1 and 3 months after discharge. ●, children with previous asthma diagnosis; ▲, children without previous asthma diagnosis

To elucidate whether AHR was enhanced in patients with A(H1N1)pdm09 infection, we measured AHR of 12 pediatric participants infected with A(H1N1)pdm09 at 1‐ and 3‐months after discharge (Table 1). PC_20_ of all patients were lower than 8,000 μg/ml at 1month after discharge. PC_20_ significantly increased at 3 months after discharge compared to that at 1 month after discharge, suggesting that AHR was enhanced in the acute phase (1 month post discharge) of these patients (Figure 1; 1 month after discharge vs. 3 months after discharge; 1036 vs. 1597 μg/ml, *p* = .009).

**Table 1:** Clinical characteristics of patients infected with A(H1N1)pdm09 at 1 month after discharge

***n* = 12**	
Age (years)^a^	7.7 (4.9–11.8)
Sex (M/F)	9/3
IgE (IU/ml)^a^	932 (124–2,680)
Inhaled allergen	
Sensitized to mite (*n*)	11
Mite IgE (UA/ml)^b^	>100 (<0.34–>100)
Sensitized to Japanese cedar (*n*)	8
Japanese cedar IgE (UA/ml)^b^	6.8 (<0.34–92)
Sensitized to *Alternaria* (*n*)	2
*Alternaria* IgE (UA/ml)^b^	<0.34 (<0.34–4.6)
Sensitized to cat (*n*)	1
Cat IgE (UA/ml)^b^	<0.34 (<0.34–7.2)
%FVC^a^	90.6 (70.0–119)
%FEV_1.0_ a	96.4 (75.8–111)
%V_50_ a	97.2 (55.5–160)
Previous asthma diagnosis	7
Asthma severity (*n*)	
Intermittent	6
Mild persistent	1
Moderate persistent	0
Severe persistent	0

Abbreviations: FVC, forced vital capacity; FEV_1.0_, forced expiratory volume in 1 s; IgE, immunoglobulin E; V_50_, maximal flow at 50% vital capacity.

^a^ Data are mean (range).

^b^ Data are medians (range).

### 3.2 Enhanced AHR in asthmatic mice with A(H1N1)pdm09 infection

**Figure 2:**
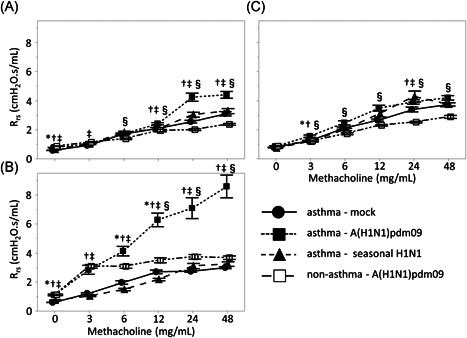
Resistance of respiratory system (R_rs_) to methacholine challenge in asthma model mice infected with vehicle (mock), A(H1N1)pdm09, or seasonal H1N1 (A/Puerto Rico) at (A) 3, (B) 7, and (C) 10 days postinfection. Data are presented as means ± standard error of the mean (*SEM*); ●, asthma–mock; ■, asthma–A(H1N1)pdm09; ▲, asthma–seasonal H1N1; □, non‐asthma–A(H1N1)pmd09. Differences between means are presented as follows: asthma–mock versus asthma–seasonal H1N1, *p < .05; asthma ‐ seasonal H1N1 versus asthma ‐ A(H1N1)pdm09, † *p* < .05; asthma–A(H1N1)pdm09 versus asthma–mock, ‡*p* < .05; and asthma–A(H1N1)pdm09 versus non‐asthma–A(H1N1)pdm09, §*p* < .05

The total airway resistance revealed by the R_rs_ value was significantly increased in the A(H1N1)pdm09 mouse group compared to that in the seasonal H1N1 mouse or control group (Figure 2); this was particularly prominent after treatment with 48 mg/ml methacholine at 3 and 7 days after infection (3 days postinfection A(H1N1)pdm09 versus seasonal; 4.40 versus 3.29 cmH_2_O.s/ml, p < .001, versus control; versus 3.11 cmH_2_O.s/ml, p < .001; 7 days postinfection A(H1N1)pdm09 versus seasonal; 8.60 versus 3.24 cmH_2_O.s/ml, p < .001, versus control; versus 3.00 cmH_2_O.s/ml, *p* < .001). However, there was no significant difference among the three groups at 10 days postinfection. In contrast, there were no significant differences in the R_rs_ between the seasonal H1N1 and mock groups at 3, 7, or 10 days postinfection.

**Figure 3:**
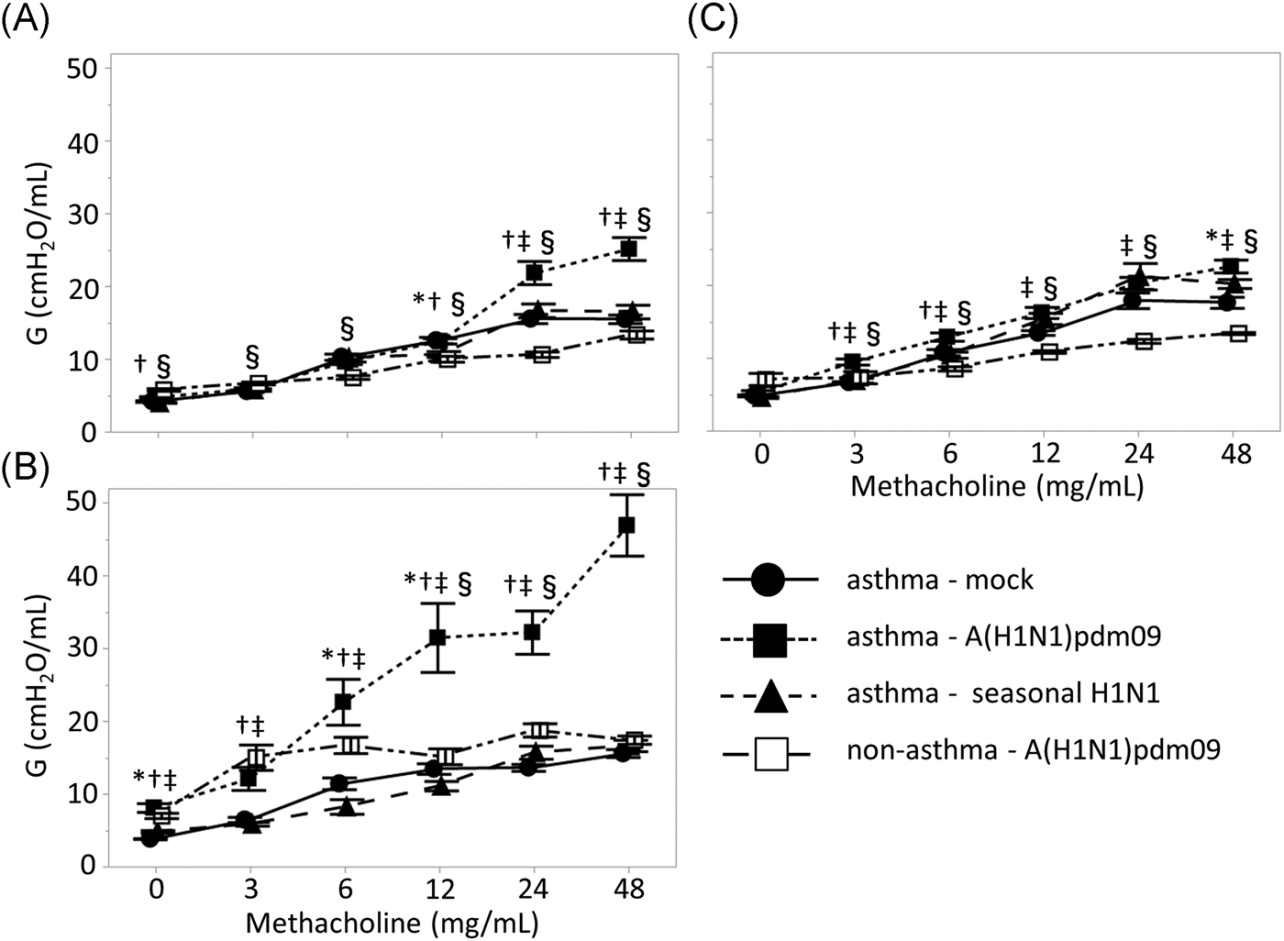
Tissue damping (G) of methacholine challenge in asthma model mice infected with vehicle (mock), A(H1N1)pdm09, or seasonal H1N1 (A/Puerto Rico) at (A) 3, (B) 7, and (C) 10 days postinfection. Data are presented as means ± *SEM*; ●, asthma–mock; ■, asthma–A(H1N1)pdm09; ▲, asthma–seasonal H1N1; □, non‐asthma–A(H1N1)pmd09. Differences between means are presented as follows: asthma–mock versus asthma–seasonal H1N1, *p < .05; asthma–seasonal H1N1 versus asthma–A(H1N1)pdm09, †*p* < .05; asthma–A(H1N1)pdm09 versus asthma–mock, ‡*p* < .05; and asthma–A(H1N1)pdm09 versus non‐asthma–A(H1N1)pdm09, §*p* < .05

Next, peripheral airway resistance as reflected by G was compared among the three groups (Figure 3). The peripheral airway resistance was also significantly increased in the A(H1N1)pdm09 group compared to that in the seasonal H1N1 and control groups; this difference was particularly prominent after treatment with 48 mg/ml methacholine at 3 and 7 days postinfection (3 days postinfection A(H1N1)pdm09 versus seasonal; 25.2 versus 16.5 cmH_2_O/ml, *p* < .001, versus control; versus 15.5 cmH_2_O/ml, *p* < .001; 7 days postinfection A(H1N1)pdm09 versus seasonal; 50.0 versus 16.7 cmH_2_O/ml, *p* < .001, versus control; versus 15.6 cmH_2_O/ml, *p* < .001). However, these differences in airway resistance were not significantly different among the three groups at 10 days postinfection.

We investigated the changes in AHR of non‐asthmatic mice with A(H1N1)pdm09 infection (Figures 2 and 3). Although AHR was enhanced with A(H1N1)pdm09 infection in non‐asthmatic mice, the changes in AHR were slight compared to the changes observed in asthmatic mice, suggesting that A(H1N1)pdm09 infection more robustly enhances AHR in asthmatic animals.

We further evaluated the alternations of AHR in asthmatic mice during the postinfection period of A(H1N1)pdm09 infection. Airway resistance was significantly enhanced at 7 days postinfection compared to at 3‐ or 10‐day postinfection (*p* < .001), whereas there were no differences in airway resistance between 3 and 10 days postinfection. When the body weight of mice was compared among the three groups to evaluate the systemic damage caused by A(H1N1)pdm09 infection at 3, 7, or 10 days postinfection, no significant differences between days were detected (data not shown).

### 3.3 Histopathological findings in the lungs

**Figure 4:**
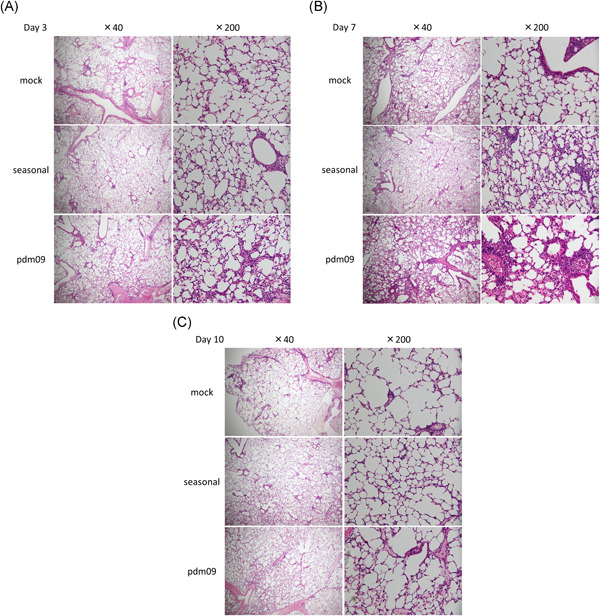
Histopathological findings after influenza infection. Photomicrographs of hematoxylin and eosin‐stained lung tissue at (A) 3, (B) 7, and (C) 10 days postinfection with mock, seasonal H1N1 (A/Puerto Rico), or A(H1N1)pdm09 influenza virus. Representative data are shown for tissues from one of four to six independent mice per group

Hematoxylin and eosin staining of lung tissues from mice at 3, 7, and 10 days postinfection is shown in Figure 4. Inflammatory cell infiltration was prominently observed in A(H1N1)pdm09‐infected mice, whereas this was rarely observed in mice infected with seasonal H1N1 at 3 days postinfection (Figure 4A). The lung inflammation was clearly observed in A(H1N1)pdm09‐ and seasonal H1N1‐infected mice at 7 days postinfection, but was more severe in A(H1N1)pdm09‐infected mice (Figure 4B). In contrast, the magnitude of inflammation was attenuated by 10 days postinfection (Figure 4C).

## 4 DISCUSSION

In this study, we measured temporal changes of PC_20_ in pediatric asthma patients with A(H1N1)pdm09 infection. PC_20_ was lowest at 1 month after discharge and significantly increased at 3 months after discharge. Furthermore, we comparatively evaluated AHR in a mouse model of asthma with either A(H1N1)pdm09 infection or seasonal H1N1 infection to investigate the pathophysiology of A(H1N1)pdm09‐infected asthmatic children. Enhanced AHR was observed in asthmatic mice with A(H1N1)pdm09 infection, which peaked at 7 days postinfection and subsequently diminished at 10 days postinfection. These results were similar to those of asthmatic children. Histopathological analysis showed that the onset of lung inflammation in asthmatic mice with A(H1N1)pdm09 infection occurred earlier and was more prominent compared to that in mice with seasonal H1N1 infection; these effects peaked 7 days postinfection and diminished by 10 days postinfection, which was consistent with the observed changes in AHR. AHR in A(H1N1)pdm09‐infected mice with or without asthma was higher than in mock and seasonal H1N1‐infected mice at 7 days postinfection. Furthermore, AHR in A(H1N1)pdm09‐infected mice with asthma was significantly higher than that without asthma. These results suggest that A(H1N1)pdm09 induces enhanced AHR as a complication of severe pneumonia, especially in mice with asthma. The desaturation and enhanced AHR observed in asthmatic patients must be induced by the pulmonary inflammation during A(H1N1)pdm09 infection, but not seasonal H1N1.

The severity of AHR reflects the inflammatory state of the airways.^19^ Several studies have reported that AHR can be enhanced by inflammatory and Th2 cytokines, such as tumor necrosis factor (TNF)‐α, interleukin (IL)‐6, and IL‐13.^20^, ^21^, ^22^ TNF‐α secreted from airway macrophages or airway epithelial cells after respiratory virus infection increases levels of adhesion molecules, such as intercellular adhesion molecule‐1 on epithelial cells, thereby inducing the recruitment of eosinophils and contributing to epithelial damage and AHR.^23^, ^24^, ^25^, ^26^, ^27^ TNF‐α is associated with wheezing in human infants.^20^ IL‐6 is secreted from epithelial cells in respiratory virus infection and induces airway inflammation and bronchospasms in patients with asthma and upper respiratory tract infections.^21^, ^24^ Our previous study showed that IL‐6 and TNF‐α levels in the bronchoalveolar lavage fluid of A(H1N1)pdm09‐infected mice were significantly higher than those in seasonal H1N1‐infected mice within 3 days after infection.^14^ Therefore, A(H1N1)pdm09 infection may enhance AHR by inducing the production of high levels of inflammatory cytokines during lung inflammation in asthmatic mice, which may also occur in human cases.

As explained above, AHR in A(H1N1)pdm09‐infected children was alleviated by 3 months after discharge compared to findings at 1 month after discharge. This finding is supported by our data in A(H1N1)pdm09‐infected mice showing that the enhanced AHR at 3 and 7 days postinfection decreased to the same level as in control mice at 10 days postinfection. Bozanich et al.^28^ reported that increased AHR observed in seasonal H3N1 influenza‐infected non‐asthmatic wild‐type mice at 4 days postinfection returned to control levels at 20 days postinfection, which is consistent with our findings. Together, these data indicate that it is pivotal to treat patients with severe asthma exacerbation in the acute phase of post‐A(H1N1)pdm09 infection. Established treatments for rescuing acute severe asthma exacerbation complicated with severe pneumonia resulting from A(H1N1)pdm09 infection have not yet been developed. We are currently investigating approaches for treating acute severe asthma exacerbation occurring with A(H1N1)pdm09 infection.

There were some limitations to this study. First, we did not evaluate AHR, inflammatory or Th2 cytokines, or virus titers in the bronchoalveolar lavage fluid at the same time. Lung tissues were collected and used for pathological analysis after AHR evaluation, as we previously reported the cytokine profiles in the bronchoalveolar lavage fluid of A(H1N1)pdm09 mice.^13^ Second, we could not evaluate AHRs of the pediatric participants before or during A(H1N1)pdm09 infection for ethical reasons to verify whether or not enhanced AHR during the infection were alleviated in the postinfection phase. Instead, we measured AHR at 1 and 3 months after discharge.

In conclusion, AHR was significantly enhanced in both pediatric asthma patients and asthmatic mice in acute phase of A(H1N1)pdm09 infection. Furthermore, A(H1N1)pdm09‐infected asthma model mice showed more severe pulmonary inflammation in the acute phase postinfection, compared to that occurring in asthmatic mice with seasonal influenza infection. Enhanced AHR subsequently returned to normal levels with the amelioration of lung inflammation, suggesting that appropriate treatment during the acute phase after A(H1N1)pdm09 infection is essential for avoiding severe respiratory conditions.

## KEY MESSAGE

Pediatric asthma patients showed enhanced signs of airway hyperresponsiveness at 1 versus 3 months post hospital discharge. This study also used mouse models of asthma to test whether pandemic or seasonal strains of influenza could enhance airway hyperresponsiveness. The pandemic A(H1N1)pdm09 strain significantly worsened airway inflammation and function compared to the seasonal flu, although these effects were limited to the acute phase at 7 days post infection. By 10 days post infection, the resolution phase was reached and differences between groups of mice infected with different influenza virus strains were negligible. In summary, our study indicates that acute influenza infection can exacerbate asthma phenotypes, particularly pandemic strains that cause severe immunopathology.

## CONFLICT OF INTERESTS

The authors declare that there are no conflict of interests.

## AUTHOR CONTRIBUTIONS

Taira Ariyoshi and Junichiro Tezuka contributed equally. Taira Ariyoshi, Hiroki Yasudo, Shouichi Ohga, and Shunji Hasegawa were the principal investigators who take primary responsibility for the study. Taira Ariyoshi, Yasufumi Sakata, Tamaki Nakamura, and Takeshi Matsushige performed the mouse experiments. Hideki Hasegawa, Noriko Nakajima, Akira Ainai, Atsunori Oga, and Hiroshi Itoh performed histopathological assays. Komei Shirabe, Shoichi Toda, and Ryo Atsuta supported this study with helpful discussions. Taira Ariyoshi, Hiroki Yasudo, Shouichi Ohga, and Shunji Hasegawa wrote the manuscript.

## Data Availability

The data that support the findings of this study are available from the corresponding author upon reasonable request.
